# Participatory urban governance: Multilevel study

**DOI:** 10.1371/journal.pone.0229095

**Published:** 2020-02-21

**Authors:** Piotr Zientara, Anna Zamojska, Giuseppe T. Cirella

**Affiliations:** 1 Faculty of Economics, University of Gdansk, Sopot, Poland; 2 Faculty of Management, University of Gdansk, Sopot, Poland; Qazvin University of Medical Sciences, ISLAMIC REPUBLIC OF IRAN

## Abstract

Constraints and stakeholder theories are used as a theoretical framework to explore civic engagement and participatory practices in cities. Based on data gathered in Gdansk, Poland, hierarchical level modelling examines the socio-psychological mechanism that underlies an individual stakeholder’s intention to participate in the operation of a facility run by a municipality-owned company. It conceptualizes this interaction as location-dependent and nested. Results indicate that stakeholder attitude to the facility and their perception of influence were—unlike their perception of voice—positively related to their intention to participate in its functioning while location proved to be negatively related.

## Introduction

It is hardly in dispute that, over the last two decades, there has been a surge of interest in the interplay between governance, participation and democracy in cities [1–13]. Participatory urban governance, conceptually rooted in the logic of pluralism [3] and the Habermasian narrative of deliberative discursive processes [14–17], emphasize inclusiveness, collaboration and consensus-seeking. Therefore, it presupposes empowerment and participation of city residents in urban decision-making processes [5,18–20]. In practice, this means giving a greater say and more power to all residents, especially to the impoverished and marginalized [6]. Recent years, for instance, have seen growing popularity of participatory budgeting [9,19] and collaborative planning [5]; however, city residents should be willing to get engaged in the first place [21]. Crucially, pluralism also lies at the core of stakeholder theory, which holds that the raison d’être of the company is to act as a vehicle for furthering the interests of its stakeholders [22]. To that end, they ought to ensure that the voice of their stakeholders is heard and that their needs are met [23]. For this to happen, it is necessary to enter into dialogue which, in turn, underlines the role of communication [24]. Even though a shareholder approach is still predominant in business praxis [25], a stakeholder perspective is gaining traction among progressive economists and academics [26]. Arguably, it should be of particular appeal to managers of municipality-owned companies, where the nominal owner is the city government elected in a democratic local election (i.e., rather than unelected shareholders). Such entities are increasingly set up with a view to operating facilities funded by the taxpayer. The Brooklyn Navy Yard Development Corporation, which operates the yard for the city of New York, is a good case in point [27]. One consequence of this is that most stakeholders of such a municipality-owned company (i.e., its employees and local customers) are, at the same time, voters qua citizens of a democratic urban polity.

Thus, in line with the logic of participatory governance and stakeholder theory, city residents as well as other stakeholders should participate in the management of a facility financed by their money and located in their space [28]. This prompt, as just mentioned, the question of whether they are willing to get engaged, with all its consequences for the dynamics of local civic life. Research shows that it is better-educated, higher-income residents who are “most likely to be strongly involved in a community’s civic life” [29]. But the fact remains that people, regardless of their level of education and income, can get engaged mainly in their free time. That, in turn, bears upon activity preferences, which is the focus of hierarchical leisure constraints theory [30]. It states that individuals form preferences for particular actions on the basis of motivators and constraints. In this sense, the conviction that one has no say or influence may act as a constraint, thereby leading them not to engage and vice versa.

When it comes to the interplay between constraints and motivators in the context of citizenry participation, in question is another factor, namely, location. This is because distance can also act as a motivator and a constraint to civic engagement. When a facility is located far away from a person’s abode or workplace and hence has only an indirect (i.e., lesser) impact on their everyday lives, they might not be particularly likely to get engaged in its construction and functioning. Conversely, when it lies nearby and hence has a direct (i.e., bigger) impact on their experience of local space, they might have a greater incentive to invest their time and to participate. The upshot is that the relationship between stakeholders and a municipality-owned company operating a publicly-funded facility might be explored through the lens of constraints theory, at the same time being conceptualized as location-dependant and as inherently nested (i.e., an individual stakeholder within a stakeholder group within a neighbourhood within a city).

In summary, at issue is a socio-psychological mechanism that underlies individual intention to participate in accordance with the main tenets of stakeholder theory and urban participatory governance. Exploration of this mechanism is particularly relevant in Eastern European countries (i.e., such as Poland), where, prior to the collapse of communism in 1989, participatory democracy, political pluralism, citizen participation and stakeholder engagement were empty words [31]. Accordingly, this study draws upon data collected among stakeholders of Miedzynarodowe Targi Gdanskie (MTG)–a company that is owned by the municipal government of Gdansk, which favours participatory forms of urban governance. MTG, unlike Brooklyn Navy Yard Development Corporation (BNYDC) [27], is a for-profit company that operates an exhibition centre called AmberExpo located in a disadvantaged district called Letnica. The construction and functioning of AmberExpo, alongside that of a football stadium, was supposed to help transform Letnica into an affluent district, which implies that its residents as well as all Gdansk inhabitants have a vested interest in its management. The focus on Letnica is not coincidental since, as noted above, there is evidence that blighted, depressed neighbourhoods are characterized by low levels of civic participation. In the light of these considerations, this study aims to answer the following research question. What is the socio-psychological mechanism that underpins stakeholder engagement in the functioning of a publicly-funded company in an ex-communist country?

A vast body of research focuses on assorted aspects and forms of participatory urban governance [1–12]. There is a growing corpus of writing on stakeholder theory in general [22] and stakeholder engagement in the development of infrastructure projects in particular [32–34]. A few studies explore different aspects of stakeholder management in public administration (e.g., de Bussy and Kelly [35]). However, less attention has been paid to the very socio-psychological mechanism that underlies an individual stakeholder’s intention to get engaged in the processes that happen in their local space (i.e., as opposed to the single-level analysis of such drivers as education and income or race). Relatedly, to the best of our knowledge, no relevant research work draws on a similar theoretical-cum-methodological approach (i.e., a combination of stakeholder and constraints theories with a multilevel analysis and hierarchical linear modelling (HLM)). In this way, this study makes a number of contributions of theoretical, practical and methodological character. The structure of the paper is as follows. The next section offers the theoretical background of the research and develops hypotheses. Subsequently, we present our research method and findings. Discussion of theoretical and managerial implications ensues. The final part highlights the study’s limitations and suggest future research avenues.

## Theoretical background and hypotheses

### Participatory urban governance

Pluralism, as mentioned in the introduction, is central to participatory governance [3,36], interest in which has been aroused by growing public disillusionment with traditional representative democracy and, in particular, the realization that its institutional forms might be increasingly inadequate for the complex realities of modern society, which sometimes results in the disenfranchisement of less wealthy residents. Arguably, it is the Habermasian theory of deliberative discursive processes that has provided much of the theoretical underpinning for participatory practices in cities. His “ideal speech situation” presupposes involvement of citizens (i.e., plural actors) in the communication process that aims at rational consensus-seeking and, ideally, leads to reconciliation of conflicting interests [15]. This logic emphasizes broad inclusiveness and collaborative effort through deliberative mechanisms which per se is deeply entrenched in left-leaning conceptualizations of citizenship and community. Thus, the literature features such overlapping notions as empowered participation, grassroots empowerment, community participation, resident involvement and citizen stakeholder engagement.

Unsurprisingly, participatory governance has become the subject of much debate. On the one hand, the accent has been on definitional-cum-terminological problems. Lyons et al. [8], for instance, speak of the “vagueness associated with the concepts of participation and empowerment […]”. Indeed, not only have questions arisen as to what participation and empowerment really mean, but also whether empowerment leads to participation or vice versa [12,19,37–39]. In other words, the question is whether giving citizens more power prompts them to participate or, conversely, whether allowing them to participate results in them being empowered. Much attention has also been paid to how participatory institutions and mechanisms should be conceived so as to engender Habermasian ideal speech situations [20]. It has been argued that community empowerment cannot be effectuated by institutions per se; what matters, above all, is a community’s ability both to mobilize resources and, crucially, to draw on local social capital, understood here as its inhabitants’ willingness to accept shared responsibility and to develop a sense of common purpose and, ultimately, to engage in urban decision-making [21]. City residents’ unwillingness to participate (i.e., civic disengagement) risks making it harder to harness local knowledge and collective action, which is widely acknowledged as one of the greatest benefits associated with this form of governance [40]. That, in turn, has led some researchers to play up the role of secondary associations [3]—such as trade unions, neighbourhood groups or local non-governmental organizations—which might intermediate between authorities and the individual. By contrast, others see government as “an enabler of civic engagement” [11].

On the other hand, criticism has been levelled at the very nature of the Habermasian deliberative process [36,41]. Most notably, Mouffe [36] doubts the fundamental premise of “ideal speech situation”, thereby questioning whether it is conducive to consensus-seeking and, by extension, reconciliation of conflicting views. In other words, unimpeded and undistorted communication—the existence of which, in reality, is highly problematic—is not enough to attain a rational consensus and, consequently, to reconcile opposing interests. Relatedly, Laclau and Mouffe [41] assert that such a consensus cannot be achieved amongst multiple actors so long as deliberative mechanisms are deeply entrenched in hegemonic social structures. Given these theoretical discrepancies, Beaumont and Loopmans [1] proposed a paradigm of “radicalized communicative rationality”, which combines “the ideals of the Habermasian ideal speech act and communicative rationality with grassroots empowerment and bottom-up processes of participation closer to the agonistic pluralism of Mouffe”.

Furthermore, it has been suggested, too, that in Eastern Europe power devolution effectuated at the beginning of the transformation process, in the early 1990s, did little to advance the ideal of citizenry empowerment. As local authorities captured power devolved from the centre, they were particularly reluctant to redistribute it down the ladder, exhibiting a strong tendency to continue to exert a hierarchical influence over local communities [42]. Others have likewise cited “power imbalances and asymmetric distributions of opportunity and resources” as undermining this form of governance [7]. Much has also been made of the chaotic nature and ineffectuality of deliberative processes, as compared with the alleged orderliness and effectiveness of centralized, hierarchical modes of governance. With these contentious issues being still debated in academia, things seem to be clearer on the ground. Indeed, evidence has mounted that municipal governments across the world, including in Eastern Europe, have been increasingly keen to adopt participatory governance practices.

### Participatory governance and project development

The very nature of participatory urban governance requires municipalities to establish mechanisms by which residents can voice their opinions and participate in decision-making. This matters both during the preliminary stages of the project, when the precise location and the construction practicalities are decided [5], and after the project’s completion when it is run by a municipality-controlled entity. Moreover, city residents must be willing to invest their time and to “deliver their input” by attending consultation meetings, posting relevant comments on social media sites or, after the project has been carried through, by participating in its management. Since this requires one to make decisions in respect of one’s (free) time, at issue are activity preferences.

In practice, however, there are a number of caveats concerning these issues. Above all, it is important to remember that any stakeholder group is composed of individuals who may have different views and, crucially, values, which define “what people believe to be fundamentally right or wrong” [43]. Also, “values conceptualize needs and desires that can be represented to other persons as valid claims” [44]. This leads them—even if they are conceived as belonging to the same stakeholder group—to frame particular plans and policies differently. It follows that there might be a divergence of interests and goals not only between different stakeholder groups, but also, crucially, within the same stakeholder group.

A study by Zientara [45] illustrates this point. In the first decade of the 21^st^ Century, residents of the same Polish locality became divided over the local government’s decision to grant a private-sector company permission to erect windmills generating clean electricity. Even though the operation of windmills is environmentally-friendly, their presence mars the aesthetic qualities of the landscape. Hence, while the former aspect pleased local inhabitants who valued environmental sustainability, the latter angered those who lived on agro-tourism. As a result, not only was there dissonance among the residents, but also contestation of a project framed by a democratically-elected government. In short, even though local decision-making was legitimized by the democratic process, the community was riven by conflict (i.e., noted in the previous section regarding the question of how to reconcile opposing interests features saliently both in the Habermasian reasoning and its critique by Mouffe).

### Implications of location for participatory governance and project development

Yet conflict within the same community can also be looked at through the prism of location (i.e., distance). In the above example, given the town in question was a small locality, most inhabitants lived near the area where windmills were planned to be erected. Thus, they were all concerned by the construction in equal measure and other factors (i.e., such as personal interests and values), rather than location itself, played a more important role in shaping their attitudes to the project. However, in a big city, where large distances are in play, location may have an impact on how residents of a particular neighbourhood perceive an infrastructure project. For example, if the erection of mobile-phone base stations is in question (i.e., allegedly producing harmful emissions), those living near the proposed site—unlike those living farther away—usually oppose the project (i.e., Nimbyism). This, bearing on the concept of scale [46], has special significance for urban decision-making and project development. Scale is defined as “the level of geographical resolution at which a given phenomenon is thought of, acted on, or studied” [47]. It is conceptualized as the local, the urban, the regional or the national. At issue, therefore, is the “nested hierarchy of bounded spaces of differing size” [48]. Nonetheless, scale—rather than being understood merely as a bounded geographical area and, by extension, as a static and self-enclosed container [49]–has come to be seen as a societal framework for a wide array of economic interactions and political contestations. Therefore, scale is conceived as being socially constructed [50]. At the same time, scale “affects city government too. Cities cannot grow beyond a certain size as free-riding city-states […]” [6].

Given that, as argued by Lefebvre [28], it is residents of a particular locality that experience local space on a daily basis, they should have an exclusive right to shape their small local reality [40]. The upshot is that it is the inhabitants of a neighbourhood—rather than decision-makers at scales other than a neighbourhood (i.e., at the level of a city at large)–that should be able to control the formation and reconfiguration of space in their area [18,29,51]. However, it seems problematic to fully agree with this logic [52]. What happens, for example, if a municipal government intends to build a road going through a particular district in order to ease congestion in the entire city—a typical urban infrastructure project financed by the taxpayer? If the government learns, having consulted residents of this particular neighbourhood, that they oppose the project, should it somehow disregard contestation and force it down the local community’s throat on the grounds that the road will serve the interests of all city dwellers? Should the narrowly-defined interests of a small constituency (i.e., those living near the proposed site) prevail over the interests of a wider public (i.e., those living farther from the project, but within the bounds of the city, but also tourists and other visitors from outside it)? On the other hand, what happens if some of the neighbourhood’s residents, nonetheless, see the sense in the construction of the road and hence do not concur with their neighbours (i.e., usually, a vocal majority) in their belief that the project ought to be scrapped or modified? Should their voice be dismissed as unrepresentative of the whole neighbourhood and sacrificed on the altar of (unconstrained) majoritarianism? Of course, such questions should be answered at the preliminary stage of a project [5]. Yet city residents should also participate in the functioning of a constructed facility, thereby interacting with a municipality-owned company that operates it. This, in turn, evokes stakeholder theory.

### Stakeholder theory and attributes

Even though stakeholder theory has been criticized for conceptual confusion and definitional vagueness [53], it has succeeded in establishing itself as an alternative managerial paradigm [22]. Given the nature of the criticism, the fundamental question concerns stakeholder identity. According to Freeman and Reed [54], stakeholders are “any identifiable group or individual who can affect the achievement of an organisation’s objectives, or who is affected by the achievement of an organisation’s objectives”. It follows that, for a stakeholder to be recognized as such, “there must be a stake, right, interest, etc.” [55]. Thus, several binary stakeholder typologies have been put forward. For instance, stakeholders are classified as “internal” and “external” [56], “primary” and “secondary” [57] or “core” and “peripheral” [58]. This is important since, in theory, organizations ought to treat all stakeholders equally [59], in accordance with the principle that “no single set of interests prevail over all others” [60]. However, in practice, “it is unlikely that all stakeholder expectations will be met” [34]; hence, the necessity of prioritizing stakeholders and, consequently, of setting the criteria for prioritization.

Mitchell et al. [61] developed a stakeholder classification based on a triplicity of power, urgency and legitimacy. Drawing on this model, Mainardes et al. [60] came up with six stakeholder types (i.e., regulator, controller, partner, passive, dependent and non-stakeholder). Miles [55] proposed a different systemization that groups stakeholders into four types (i.e., influencers, claimants, recipients and collaborators). Mitchell et al.’s framework has also underpinned attempts at stakeholder impact analysis in the context of project development. These include the vested interest-impact index [62], the external stakeholder impact index [63], the power-interest matrix [64] and the power-predictability matrix [65]. Also worth mentioning is the framework developed by McElroy and Mills [59] which focuses on the extent to which stakeholders back or oppose a given initiative, as manifested in their “position towards the project”.

In the light of these considerations, it is possible to identify a set of stakeholder attributes that encompasses—alongside Mitchell et al.’s power, legitimacy and urgency—influence, impact, interest, attitude and support [66]. Arguably, of special significance are the notions of power and closely related influence and impact. Mitchell et al. [61] define power as the ability to “gain access to coercive, utilitarian, or normative means, to impose its will in a relationship”. Specifically, stakeholders may derive their power from their capacity both to withdraw resources from the organization [63] and, critically, to mobilize political forces or public support for their cause, which is relatively unproblematic in the era of social media.

Power, being central to the stakeholder-organization relationship, bears on the issue of governance and, by extension, on the notion of “stakeholder democracy” [67]. Conceptually rooted in a Habermasian deliberative, inclusion-oriented approach [14,16], it holds that, since shareholder interests—which are manifested in the managerial pursuit of shareholder value maximization [26]–should not prevail over the interests of other stakeholders, management should not have a monopoly on organizational decision-making. It follows that a managerial “dictatorship”, which relies on a single source of authority and power (i.e., managers qua shareholder representatives in line with the main tenet of shareholder theory), should be replaced with a more democratic, inclusive, collaborative and consensus-seeking arrangement. Yet, for this to work, not only should managements be disposed to share power—which might be less problematic at city government-controlled entities than at organizations with private ownership—but also stakeholders should be willing to get engaged and exercise their right to a greater say in how the organization is run. Since that concerns one’s motivation and, at the same time, entails making decisions regarding one’s time, at issue are activity preferences.

### Constraints theory and stakeholder engagement

This aspect is the focus of hierarchical leisure constraints theory [30], which holds, as mentioned in the introduction, that people form preferences for particular actions on the basis of motivators and constraints. In other words, when an individual considers whether to get engaged in an activity or not, the interplay of motivators and constraints is instrumental in determining their behaviour. Specifically, structural as well as interpersonal and intrapersonal constraints underpin an attitude and, ultimately, one’s intention to behave in a particular way. There is growing recognition that the theory, which originally served to explain leisure-related preferences, can be “applicable to a variety of human behaviours” [68]. Admittedly, this is also true of the theory of planned behaviour (TPB) [69]. Yet, given that this study’s focus is on a stakeholder’s activity preferences, we decided to explore these issues through the prism of constraints theory. Thus, while adhering to the main tenet of the TPB (i.e., an intention is a major predictor of one’s behaviour), we propose to see a combination of motivators and constraints as underpinning a stakeholder’s intention to participate.

In this context, the question arises of how we conceptualize the motivators and constraints related to a stakeholder’s intention to get engaged in the functioning of a facility run by a municipality-owned company (i.e., our dependent variable). Having reviewed the literature on stakeholder attributes and engagement within the context of project development, we came up with three constructs (i.e., independent variables): attitude, influence and voice (i.e., at an individual level or Level 1). Since the same phenomena can be positively and negatively valenced [70], we assume that attitude, influence and voice can be simultaneously conceived as a motivator and a constraint, depending on whether an individual stakeholder holds a positive or negative attitude, and believes that they have some influence and that their voice counts [71].

In fact, the following psychological mechanism is at work: if one holds a negative attitude and believes that they have no, or too little, influence or that their voice is not taken into account, they may decide that there is no point in getting involved (i.e., constraint). Conversely, if they hold a positive attitude and believe that they have (at least, some) influence and their voice matters (at least, to some extent), they may be incentivized to act (i.e., motivator). It is possible to draw analogies between our reasoning and the logic that applies to the mechanism by which the polls affect how individuals vote: “Some people won’t bother to vote because they live in a safe seat, whereas others may make an effort to vote if polls suggest the result in their constituency will be close. Then, by extension, small or new parties may not get support if the big parties dominate the polls and potential voters think their vote will therefore be wasted on the newcomer” [72].

The implication is that the same construct—depending on how it is perceived—can be, at the same time, a motivator and a constraint, thereby acting as an encouragement or discouragement to participation. Hence the following hypotheses:

*Hypothesis 1 (H1)*: *There is a positive relationship between a stakeholder’s attitude and their intention to participate*.
*Hypothesis 2 (H2)*: *There is a positive relationship between a stakeholder’s perception of their influence and their intention to participate*.
*Hypothesis 3 (H3)*: *There is a positive relationship between a stakeholder’s perception of their voice and their intention to participate*.

Yet, as noted earlier in the text, there is another factor—location (i.e., distance)–that comes into play. Likewise, it can act both as a motivator and a constraint. When a facility is situated far away from a person’s residence and hence has only an indirect (i.e., lesser) impact on their everyday lives, they might not be particularly likely to participate in its construction and functioning (i.e., constraint). Conversely, when it is located nearby and hence has a direct (i.e., bigger) impact on their experience of local space, they might have a greater incentive to invest their time and to engage (i.e., motivator). Put another way, interest in a particular facility naturally diminishes with distance. In fact, people tend to be less interested in what occurs relatively far from their place of residence or work, which decreases their motivation to get engaged. The above example of neighbourhood residents’ opposition to the construction of a base station also illustrates this point: the closer a planned project is located to one’s abode, the more willing one might be to participate in the debates on its raison d’être and, in all likelihood, to oppose it. It is, therefore, possible to presume that location is negatively related to a stakeholder’s intention to get engaged. Hence the following hypothesis:

*Hypothesis 4 (H4)*: *There is a negative relationship between location (i*.*e*., *distance from one’s place of residence or work to the facility) and a stakeholder’s intention to participate*.

**Fig 1:**
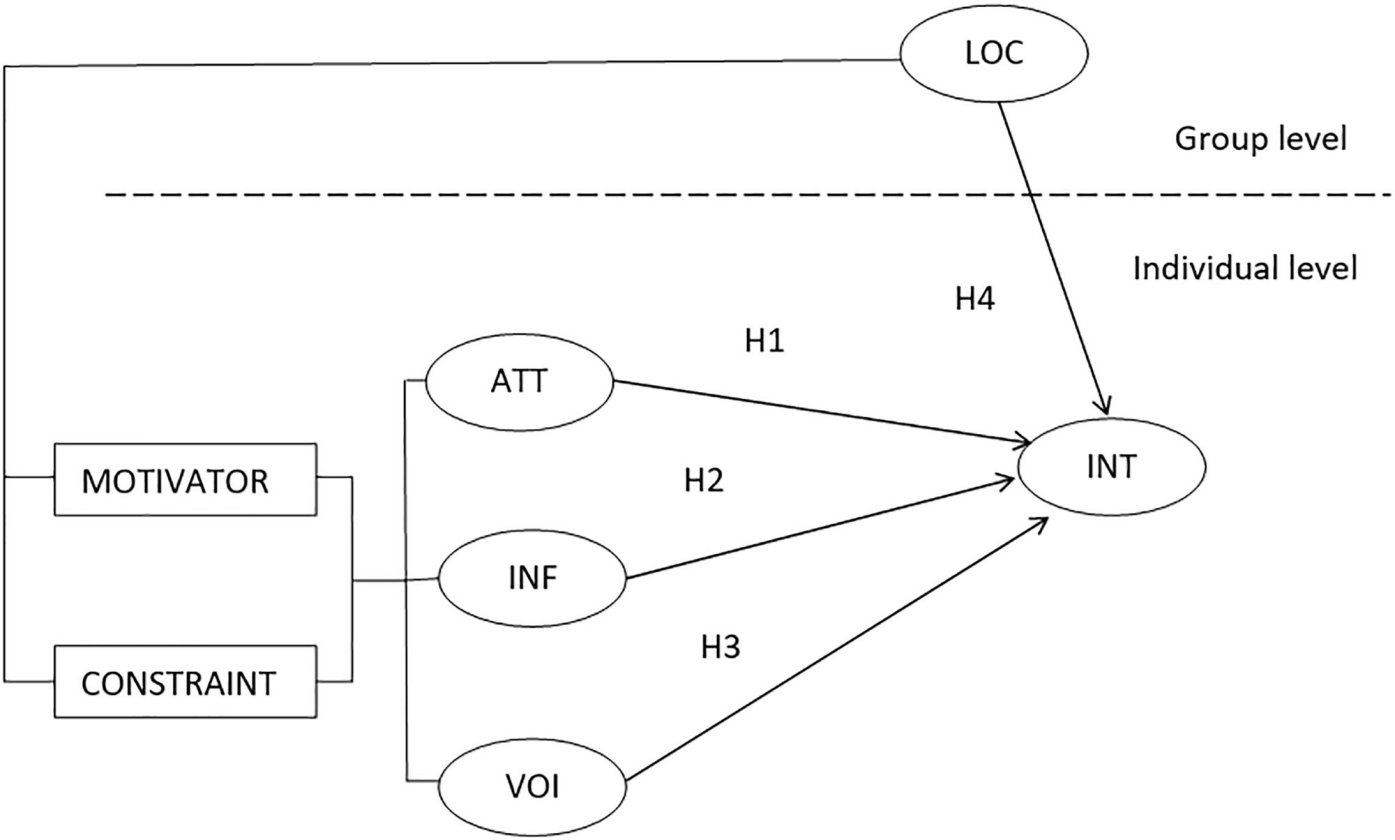
The conceptual model.

Fig 1 presents our conceptual model.

## Methods

### Context of the study

Gdansk is one of Poland’s biggest cities and an important sea port. Before 1989, ship building and seaborne trade were the mainstay of the area [42]. The city was home to the shipyard that became the cradle of the “Solidarity” trade-union movement, which precipitated the collapse of the communist regime, under which participatory democracy and stakeholder engagement were empty rhetoric [31]. After 1989, the local economy has successfully diversified into tourism and high-tech services—a process that was helped by Poland’s accession to the European Union (EU) in 2004. Since then, local authorities, capitalizing on the availability of EU funds, have carried through several large-scale infrastructure projects, including the construction of a big exhibition centre called AmberExpo. The facility, endowed with three exhibition halls and four conference halls, is located in Letnica, a disadvantaged neighbourhood. Prior to the construction of the football stadium and of AmberExpo itself, Letnica had been a byword for social marginalization and underdevelopment. Thus, both projects were meant to help transform its fortunes by attracting outside investors and stimulating local small entrepreneurship. The focus on Letnica is not coincidental since it is well-established in the literature that blighted, depressed neighbourhoods are characterized by low levels of civic participation [29]. The centre is operated by MTG—a company owned by the municipality, which is headed by the mayor (known in Polish as “president”), who cooperates with the city council. Both the mayor and the councillors are directly elected by city residents every four years. The municipality favours participatory forms of urban governance. For example, it encourages residents to participate in municipal budgeting; an initiative known as “citizen budget” allows all inhabitants to select projects that will be implemented in their neighbourhoods, thereby empowering them to shape their local space.

We employed HLM since it allows for the complex form of ordinary least squares regression. This is used to “analyse variance in the outcome variables when the predictor variables are at varying hierarchical levels” [73]. According to Dempster et al. [74] and Raudenbush and Bryk [75], the basic procedures for estimation and inference with HLM for multivariate data analysis are in line with research that centres on level-based predictors as well as value intervals and robust standard estimators. Taking this into account, we use HLM to analyse relationships between variables for different (groups of) stakeholders (i.e., individuals and firms) finding themselves at a different level. A hierarchical level of nesting, similar to research conducted by Woltman et al. [73], characterises location as being at the top-tier level (i.e., Level-3). As such, differences in data type, when applied to HLM, allow for shared variance in the hierarchical structuring of data (Table 1). All this makes HLM suitable for nested hierarchical analyses [73–76].

**Table 1:** Characteristics at each hierarchical level of the study, adopted from Woltman et al. [73].

Hierarchical level	Criteria for assignment to a particular hierarchical level
Level-3	Location level—distance from MTG
Level-2	Stakeholder group level—belongingness to one of the eight groups
Level-1	Individual level—firms (represented by individual managers) and residents who are stakeholders of MTG

### Sample and data collection

A self-report paper-and-pencil questionnaire was used to collect data from different MTG stakeholders belonging to the following stakeholder groups: (G1) firms located at AmberExpo, (G2) firms located in Gdansk, (G3) firms located in Letnica, (G4) residents of Gdansk, (G5) residents of Letnica, (G6) employees of MTG, (G7) visitors (i.e., only from outside Gdansk) at an event “FIT Festival” held at AmberExpo in February 2016 and (G8) firms (i.e., only from outside Gdansk) participating in “FIT Festival”. It is important to emphasize that stakeholders were grouped by location. In other words, the main criterion for assignment to a particular group (Table 1) was the distance from one’s place of residence or work to AmberExpo. It should also be noted that, in the case of firms, questionnaires were completed by their representatives (i.e., usually managers).

During the entire process—i.e., preparation (including a pilot test), completion and management of the questionnaires and gathered data—the research complied with the University of Gdansk’s Code of Ethics and was approved by the Institutional Review Board of the Department of Management at the University of Gdansk (IRB). As such, respondents were not misled or forced in any way to participate in the questionnaire survey (i.e., we acted on verbal consent from participants, as determined by the IRB). Moreover, respondents were beforehand informed of the nature and purpose of the research and of any expected inconveniences related to their participation.

A total of 820 responses were collected, of which 23 were deemed incomplete. Consequently, 797 responses were used for further analysis. Our sample was 57% female. Further, 37% of respondents had a university education and 63% a high-school education (S1 Table). Regarding firms, firm size was broken down into four tiers: 1–4 employees (54%), 10–49 employees (19%), 50–249 employees (21%) and 250 and over (7%). As for firm age, 78% were ten years old and 22% were more than 11 years old. These two types of stakeholder groups (i.e., residents, as broken down by age, sex and education, and firms, as broken down by firm size) constitute *different* entities and, as such, are characterized by *different* types of control variables. This means that different (types of) controls cannot be used in one and the same model.

The sampling procedure varied depending on stakeholder group. Specifically, as for G4 and G5, we used a two-stage approach. The first stage consisted in randomly selecting a street. At the second stage, systematic sampling was applied to select a flat (i.e., every fifth flat). As for (2), we randomly selected companies from a register, known as National Official Register of the Territorial Division of the Country (TERYT). As for G1 and G3, all firms based on TERYT were included (i.e., full sample). The same applies to G8. As for G6, systematic sampling was used (i.e., every second employee). Finally, we applied accidental sampling to G7.

### Measurement and statistical analyses

Having analysed extant work that discusses stakeholder attributes and draws on stakeholder surveys [32,33,62,66], we pieced together 13 items with which to measure our constructs (i.e., variables). Specifically, attitude (i.e., ATT) was measured with three items (α = 0.82); the same goes for influence (i.e., INF) (α = 0.85) and voice (i.e., VOI) (α = 0.89). Intention (i.e., INT) was measured with four items (α = 0.91). A full list of the items used in the research instrument can be found in Table 2.

**Table 2:** List of the items used in the research instrument.

Variable	Abbreviation	Item
Attitude	ATT	“I have a positive view on the functioning of AmberExpo”
		“I regard the functioning of AmberExpo as positive”
		“I believe that the functioning of AmberExpo is beneficial”
Influence	INF	“I have effective influence over the functioning of AmberExpo”
		“I am in a position to influence the functioning of AmberExpo”
		“My influence over the functioning of AmberExpo is significant”
Voice	VOI	“My opinion is taken into account when decisions are made regarding the functioning of AmberExpo”
		“My voice counts when decisions are taken regarding the functioning of AmberExpo”
		“When decisions are taken regarding the functioning of AmberExpo, my voice is not disregarded”
Intention	INT	“I am willing to participate in the functioning of AmberExpo”
		“I am willing to devote a lot of time and energy to the functioning of AmberExpo”
		“I consider myself willing to get engaged in the functioning of AmberExpo”
		“Those running AmberExpo can count on my engagement”

Finally, location (i.e., LOC) was operationalized by the distance from a stakeholder’s place of residence or work to AmberExpo. To that end, we assigned values of 0 to 3. In particular, 0 indicated stakeholders located at AmberExpo (i.e., G1 and G6), 1 –in Letnica (i.e., G3 and G5), 2 –from outside Letnica, but within the bounds of Gdansk (i.e., G2 and G4) and 3 –from outside the bounds of Gdansk (i.e., G7 and G8). The means, standard deviations, reliabilities and inter-correlations among the variables are presented in Table 3.

**Table 3:** Mean, standard deviation, reliability and Spearman correlation of individual level variables^†^.

Variable	INF	VOI	ATT	INT
INF	**(0.85)**			
VOI	0.525*	**(0.89)**		
ATT	0.485*	0.635*	**(0.82)**	
INT	0.583*	0.472*	0.599*	**(0.91)**
Mean	1.72	2.26	2.67	2.20
SD	0.83	1	1.07	1.02

^†^ n = 797; individual-level scale reliability in bold along diagonal

* p < 0.05

Data characteristics were also closely cross-referenced for level-based predictors using standardised statistical estimators. The data generating process entailed calculation of descriptive statistics for each attribute, in each of the eight groups of stakeholders, followed by the goodness-of-fit Jarque-Bera test. The Jarque-Bera test was used to check normality of the distribution of observations (i.e., based on sample skewness and kurtosis) in which Jarque’s [76] formulation (Eq 1) is tested for *v*_*1*_, … *v*_*N*_ observations over a normal distribution curve.

$$JB=\frac{N}{6}\;(W^{2}+\frac{{\left(K-3\right)}^{2}}{4})$$ (1)

Where: *N* = number of observations, *W* = sample skewness and *K* = sample kurtosis. For the sample data, we compared the four variables (i.e., ATT, INF, VOI and INT) for the two stakeholder types: firms (i.e., groups: G1, G2, G3 and G8) and residents (i.e., groups: G4, G5, G6 and G7) (Table 4).

**Table 4:** Descriptive statistics and goodness-of-fit Jarque-Bera test of the variables.

Variable	Group		N	Mean	SD	Min	Max	Skewness	Kurtosis	JB^†^
ATT	Firms	G1	12	3.94	3.00	5.00	0.75	0.02	-1.38	0.95
		G2	39	2.08	1.00	5.00	1.22	0.82	-0.42	4.60
		G3	37	2.95	1.00	5.00	1.43	-0.13	-1.46	3.42
		G8	65	3.34	1.00	5.00	1.09	-0.19	-0.79	2.05
	Individuals	G4	400	2.49	1.00	5.00	0.87	-0.33	-0.52	11.83*
		G5	113	2.83	1.00	4.67	1.05	-0.24	-0.77	3.85
		G6	25	4.32	3.00	5.00	0.63	-0.59	-0.35	1.58
		G7	106	2.35	1.00	4.33	0.95	0.24	-0.92	4.76
INF	Firms	G1	12	2.44	1.00	4.00	1.19	0.22	-1.72	1.58
		G2	39	1.46	1.00	3.67	0.77	1.51	0.91	16.10*
		G3	37	1.61	1.00	5.00	0.92	1.95	4.18	50.39*
		G8	65	2.51	1.00	5.00	1.07	0.25	-0.92	2.97
	Individuals	G4	400	1.59	1.00	4.00	0.66	1.40	1.24	156.6*
		G5	113	1.76	1.00	3.33	0.83	0.48	-1.40	13.48*
		G6	25	2.60	1.00	4.33	0.75	-0.11	0.21	0.10
		G7	106	1.54	1.00	4.00	0.77	1.54	1.45	51.18*
VOI	Firms	G1	12	2.83	1.00	4.00	1.09	-0.80	-0.73	1.55
		G2	39	1.62	1.00	4.00	0.94	1.09	-0.34	7.96**
		G3	37	2.86	1.00	5.00	1.50	-0.26	-1.69	4.83
		G8	65	3.13	1.00	5.00	1.20	-0.15	-1.04	3.18
	Individuals	G4	400	2.02	1.00	5.00	0.76	0.87	1.16	72.53*
		G5	113	2.80	1.00	4.67	0.93	-0.30	-0.60	3.39
		G6	25	2.93	1.00	5.00	1.03	0.06	-0.16	0.04
		G7	106	1.88	1.00	3.67	0.74	0.54	-0.55	6.52*
INT	Firms	G1	12	3.75	2.25	5.00	0.88	-0.53	-0.91	0.97
		G2	39	2.01	1.00	5.00	1.12	0.88	-0.04	5.00
		G3	37	2.54	1.00	5.00	1.35	0.24	-1.39	3.34
		G8	65	2.93	1.00	5.00	1.01	-0.10	-0.83	1.96
	Individuals	G4	400	1.82	1.00	4.25	0.70	0.64	-0.26	28.30*
		G5	113	2.26	1.00	4.00	1.01	0.27	-1.10	7.14**
		G6	25	4.09	2.25	5.00	0.79	-0.80	0.49	2.91
		G7	106	2.44	1.00	5.00	0.98	0.24	-0.85	4.20

^†^ Jarque-Bera test

* p < 0.05;

** p < 0.10

The results of the Jarque-Bera test allowed us to select an appropriate—i.e., parametric or non-parametric—test; the former in the case of the normal distribution and the latter otherwise (Table 5).

**Table 5:** Results of the tests for the differences between means of the groups.

Variable	Firms		Residents		
	Number of employees		Sex	Age	Education
	Parametric^1^	Nonparametric^2^	Nonparametric^3^	Nonparametric^2^	Nonparametric^2^
ATT	2.851*		0.987*	21.701*	2.866
INF		12.705*	-0.332	9.547*	1.687
VOI	2.392*		-1.378	11.623*	0.500
INT	2.917*		-0.882*	13.224*	2.260

^1^ ANOVA test;

^2^ Kruskall-Wallis test;

^3^ Mann-Whitney U test

* p < 0.05

Table 5 shows the results of the tests we performed to compare the differences between means of the groups. Regarding the firms, there were significant differences in the average level of variables (see also relevant details in Table 4). Regarding the residents, there were no education-related differences (i.e., as indicated by the absence of asterisks); by contrast, there were age-related significant differences between groups of residents. Sex-related differences concerned two variables: ATT and INT.

## Results

While performing our analysis, we used HLM 7, a computer program, and followed the guidelines on hierarchical linear modelling [77]. The first step consisted in estimating a level-one null model with no independent level-one variables. The second step involved estimating a random coefficient regression model. We then estimated an intercepts-as-outcomes model to test the direct effect of the group level variable (i.e., LOC) on INT. Table 6 shows the results of hierarchical linear modelling.

**Table 6:** Results of hierarchical linear modelling for INT†.

Variable	Null model	Random coefficient regression model	Intercepts-as-outcomes model (group level)
Level 1 (individual)			
Intercept	2.51* (0.42*)	2.52* (0.86*)	2.52* (0.30*)
ATT		0.57* (0.33*)	0.54** (0.33*)
INF		0.44* (0.14*)	0.46* (0.14*)
VOI		-0.07 (0.07)	-0.07 (0.07)
Level 2 (group)			
LOC			-0.70*
Level-1 residual variance	0.700	0.282	0.234
Likelihood ratio test χ^2^(p)	—	393.29*	399.55*
Model deviance	1192.59	799.72	705.29

^†^ n1 = 797; n2 = 4. Entries are estimates of the fixed effects (γ_s_) with robust standard errors. Estimations of the random variance components (τ_s_) are in parentheses. The τ_s_ for the intercepts also represented the between-group variance in INT and the between-group variance in INT.

* p < 0.05;

** p < 0.10

### Level-one NULL model

First, we set out to ascertain whether there was significant between-group variance in the dependent variable. To that end, we estimated a null model with no independent level-one variables. The program generates a chi-square statistic, which tests the significance of the between-group variance. In our study, the significant chi-square value (i.e., 29.15) means that INT could potentially be explained by the percentage of the total between-group variance (i.e., intraclass correlation coefficient (ICC)(1), which denotes this percentage, indicates the amount of variance that could potentially be explained by a level-two variable). Thus, an ICC(1) value of 0.32 suggests that 32% of the total variance in INT was explained by LOC, while 68% by individual factors. This implies that the groups in question differed from each other in terms of INT, which, in turn, provides justification for including LOC at the group-level analysis.

### Random coefficient regression model

Next, we sought to find out whether there was significant between-group variance in the intercepts. For that purpose, we estimated a random coefficient regression model. To confirm that the variance in the intercepts for INT—after inclusion of the predictors (i.e., ATT, INF and VOI)–is significant, we conducted a chi-square test. The results indicate that it was justified to include ATT, INF and VOI into the model (i.e., χ^2^ = 393.29, p < 0.05). We used a t-test to assess the significance of mean of the slopes across groups. This offers evidence of whether the pooled level-one slopes between the independent variables and the dependent variable differ from zero. Therefore, this test assessed whether the relationships between ATT and INT, INF and INT, and VOI and INT were significant and, by implication, whether H1, H2 and H3 were supported. The results show that H1 (i.e., t = 3.09, p < 0.1) and H2 were supported (i.e., t = 5.49, p < 0.05) and that H3 was unsupported (i.e., t = -1.24, p > 0.1).

### Intercepts-as-outcomes model

**Fig 2:**
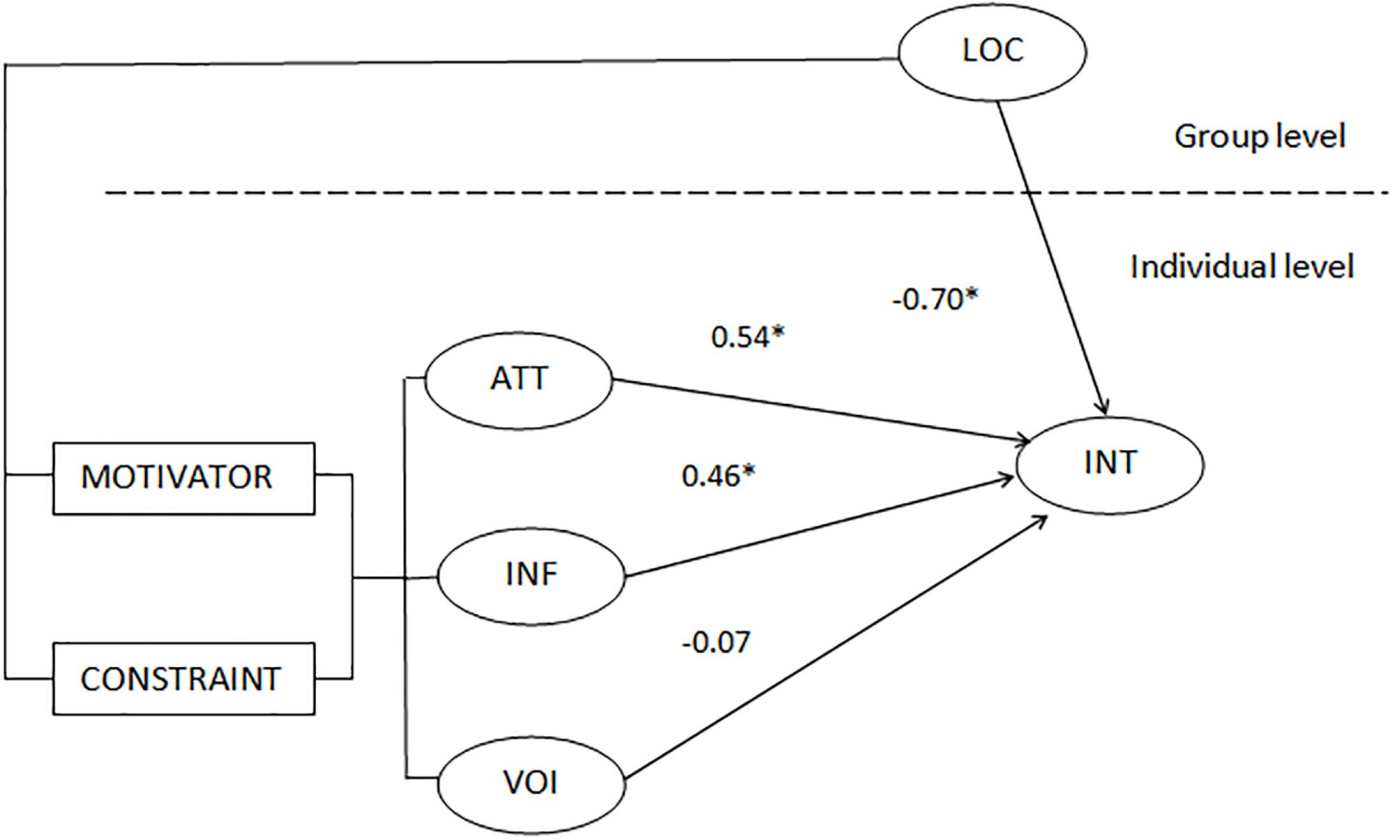
The estimated multilevel model.

To test H4, we estimated an intercepts-as-outcomes model (Fig 2). The results show that the group-level variable (i.e., LOC) has a statistically significant negative effect on INT, thereby supporting H4 (i.e., t = -13.15, p < 0.01). To confirm that the variance in the intercepts for INT across stakeholder groups is significant, we conducted, again, a chi-square test (i.e., if it turned out that there was no significant between-group variance, then a group effect would not exist). The results indicate that there was significant between-group variance (i.e., χ^2^ = 399.55, p < 0.01).

We also controlled for age, gender and education (i.e., in the case of individual stakeholders) and company size (i.e., the number of employees). Mann-Whitney U tests show that respondents aged over 41 were more willing to participate, but held a less positive attitude to AmberExpo and believed that they had less voice than those who were younger. There were no differences between men and women, and those with a university education or those with a high-school diploma in terms of ATT, INF, VOI and INT. Accordingly, these findings do not add, as much earlier research does, substance to the view that there is a positive relationship between education level and civic engagement (i.e., better-educated citizens are more likely to get involved in local civic life). Unfortunately, we did not control for income, which could also have proved revealing. In regards to firms, representatives of bigger companies (i.e., those employing more than 50 people) believed that they had less voice and less influence than their counterparts from smaller entities.

## Discussion

### Theoretical implications

This research has focused on the socio-psychological mechanism underlying a stakeholder’s intention to participate in the functioning of a publicly-funded facility run by a city-owned company within the context of participatory urban governance. Its primary contribution, therefore, lies in recognizing that, while analysing resident involvement in broadly-defined urban-managerial processes, special attention needs to be paid to the factors that determine individual willingness to participate (i.e., what drives citizen participation). Accordingly, it suggests that research on the theoretical underpinning for participatory governance, the institutional mechanisms facilitating citizen engagement or the outcomes of particular participatory forms, needs to be complemented with the study of individual motivation. At the same time, it demonstrates that constraints theory can provide a lens through which to explore this issue, which per se implies that this theory can be applied to phenomena other than leisure-related preferences. It follows that constraints theory is needed in urban scholarship because it addresses the fundamental problem, namely, what motivates people to participate in the managerial processes that concern their cities. In other words, its application helps shed new light on the socio-psychological mechanism of civic engagement. This is of great importance since, after all, giving residents a greater say in how their city is managed (or in how facilities financed by their money are run) might be seen as problematic if they are not willing to participate in the first place. Thus, the study contributes to the debate on how to advance the ideals of empowerment and participation. In so doing, it shifts the focus onto the level of the individual stakeholder who, crucially, is a member of a particular stakeholder group and a particular constituency and, at the same time, lives or works in a particular place that is located close to or far from a particular development project which per se points to the nested nature of the relationship.

In this sense, application of constraints theory as well as a multilevel approach deepens our understanding of the inherently complex realities of urban participatory processes. In fact, given that H4 was supported, it is both justified and informative to adopt a multilevel approach: a single-level approach would have failed to reflect the nested character of stakeholder identity and the location-dependent nature of the stakeholder-organization relationship. Thus, support for H4 means that location (i.e., distance) is an important factor that needs to be taken into account when analysing the mechanisms underlying participatory-governance processes in cities. Since H1 and H2 –as well as H4 –were supported, the study specifically shows that the same phenomenon (i.e., variable), depending on how it is perceived by an individual stakeholder, can be positively or negatively valenced and can, therefore, be conceived as being a constraint or a motivator. The fact that H3 was unsupported is, admittedly, hard to explain, but overall does not detract from the value of the analysis. This means that much hinges on how a person perceives and interprets particular phenomena. Another implication is that not only distinct phenomena, as is widely acknowledged, can act as constraints or motivators.

On the other hand, our findings indirectly advance the argument in favour of the adoption of a stakeholder approach by all companies. Even though, as already noted, most managers and economists still adhere to a shareholder perspective [25], there is a growing realization of the need to embrace the concept of “prosocial identity” [78], which is associated with the idea of corporate social responsibility, itself rooted in stakeholder theory and business ethics. As the number of city-owned companies (e.g., such as MTG and BNYDC) increases across the world, there is ample scope for demonstrating the merits of a stakeholder approach [22] in general and organizational pluralism in particular, whereby—through more democratic, inclusive and collaborative arrangements—the voice of other constituents is taken into account and their needs are met. This, in turn, might help the simultaneous pursuit of economic prosperity, societal fairness and environmental sustainability, a responsibility which seems to increasingly fall to the cities.

### Practical implications

**Fig 3:**
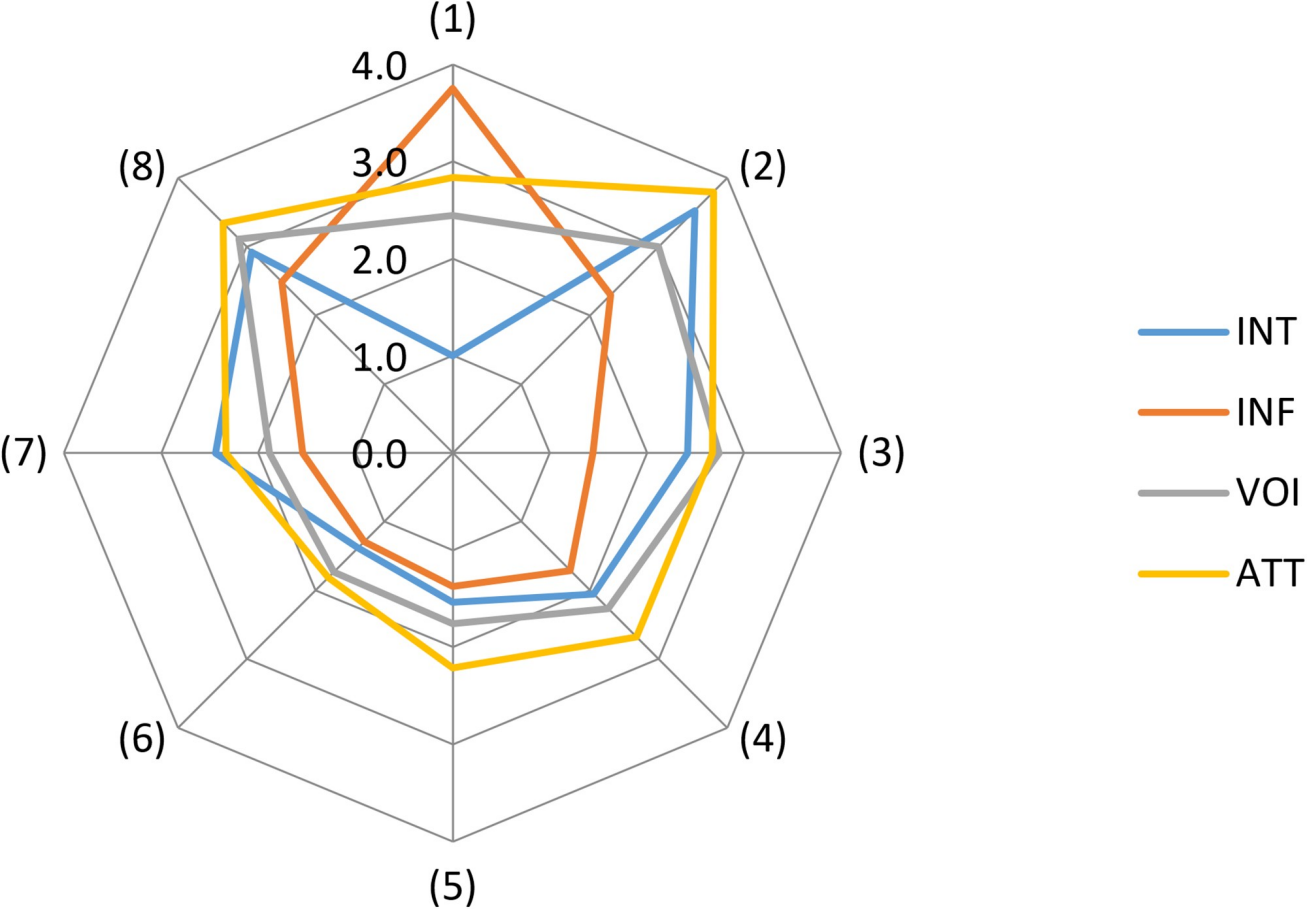
Mean of the four variables for all stakeholder groups.

**Fig 4:**
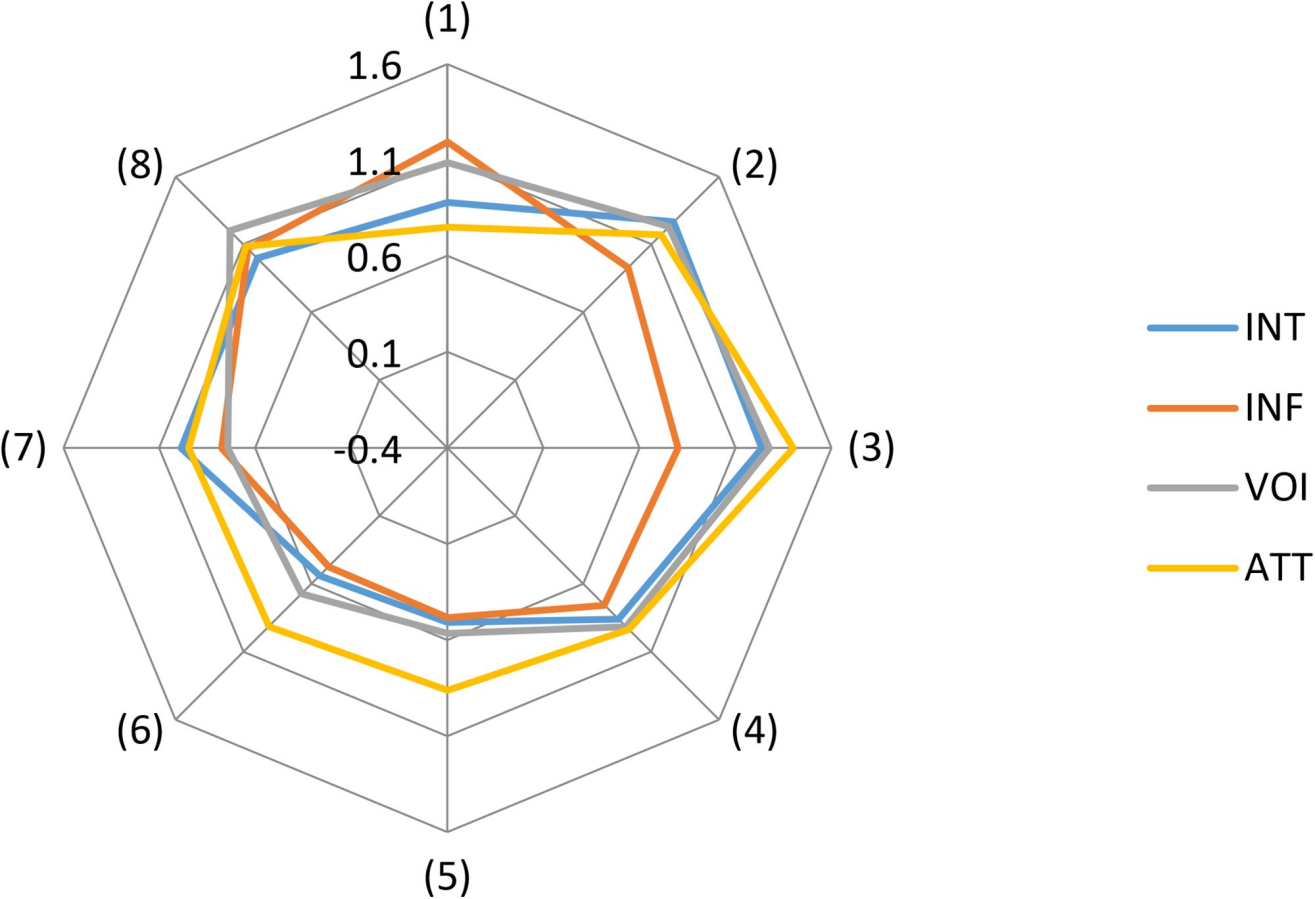
Standard deviation of the four variables for all stakeholder groups.

Our research also suggests, as Figs 3 and 4 show, that stakeholders theoretically belonging to the same stakeholder group might be characterized by a relatively high degree of heterogeneity, implying a measure of disagreement (i.e., a situation that is potentially prone to conflict). This finding has especially important implications for municipalities planning to carry out controversial projects or, in other words, projects that are likely to be opposed by many residents. It follows that urban decision-makers should remember that stakeholders in the same neighbourhood may, in essence, differ in terms of views and interests to a far greater extent than is usually assumed and that well-organized and vocal resident groups need not necessarily represent the position of all stakeholders in a given neighbourhood. Ignoring this, in turn, risks entrenching majoritarianism, which by itself is likely to engender a sense of unfairness amongst those whose minoritarian position was disregarded (following Mouffe’s line of argumentation, one might ask whether the Habermasian ideal of constraint-free communication would help achieve a consensus-based solution in these particular circumstances).

Also worth mentioning is that it was firms located at AmberExpo (1) that believed that they had the greatest influence on the functioning of AmberExpo (Figs 3 and 4). But this stakeholder group was also characterized by the greatest heterogeneity, as measured by standard deviation in this respect (which again adds substance to the view that stakeholder groups are not homogenous). By contrast, employees of MTG (6), somehow surprisingly, thought that they had the least influence (a finding that should provide food for thought for MTG management). As for VOI, it was firms located in Gdansk (2) and firms participating in “FIT Festival” (8) that believed that their voice mattered most (but, again, these groups were marked by a high degree of heterogeneity). As regards ATT, it was firms in Gdansk (2) and firms participating in “FIT Festival” (8) that constituted the stakeholder groups with the most positive attitude to AmberExpo with a modest degree of heterogeneity. Finally, it was, once again, firms in Gdansk (2) that were most willing to participate in the functioning of AmberExpo (but with a high degree of heterogeneity). Worryingly, Figs 3 and 4 show, too, that it was residents of Gdansk (4) and residents of Letnica (5), alongside employees of MTG (i.e., the vast majority of whom are also residents of Gdansk and/or Letnica) and firms located in Letnica (3), that were least willing to participate in the functioning of AmberExpo. Likewise, their attitudes to AmberExpo were not particularly positive. Likewise, their attitudes to AmberExpo were not particularly positive. These findings are consistent with previous research that demonstrates that those living in depressed, blighted neighbourhoods do not tend towards civic participation [29].

Arguably, all this—coupled with the aforesaid low participation rates in the “citizen budget” initiative—can be interpreted as the lingering legacy of the communist past. Before 1989, the Communist Party had monopoly on power, which, by definition, ruled out a multi-party democracy, participatory governance, grassroots initiative and citizen involvement [31]. The regime, despite government propaganda, was an antithesis of political plurality and stakeholder empowerment. Since the state was conflated with the Communist Party, many Poles frowned upon civic engagement on ideological grounds. All of which, and given that H1 and H2 were supported, only reinforces the case for stepping up efforts to incentivize residents of Gdansk to participate in the broadly-defined management of their city. In this sense, we concur with Sirianni [11] who argues that “government needs to become a much more strategic, systematic, and effective enabler of civic engagement […]”. Yet, in so doing, municipal authorities ought to bear in mind that effecting societal change is usually a long and complex process.

In practice, that would require mounting smart awareness-raising campaigns, in the real world and in cyberspace, explaining the rationale behind citizenry participation. Intense effort should be put into reaching out not only to inhabitants of well-to-do districts, but, above all, to residents of disadvantaged neighbourhoods, where years of marginalization and impoverishment had weighed on the collective mind-set. In this way, extolling the merits of civic engagement might help break with the legacy of the not-so-distant past. However, such public-relations moves would have to be accompanied by the introduction of new participatory practices and deliberative mechanisms. For instance, a local inhabitant could be nominated to the board of a municipality-owned company, such as MTG (i.e., guaranteed stakeholder representation). Modelled on the German practice of worker representation on company boards and in line with the spirit of stakeholder democracy, that could strengthen residents’ sense that their voice does count and that they have real influence over matters concerning their space. At the same time, much more should be done to project a “citizen-friendly” image of the city government. It follows that it ought to try to shake off its (i.e., partly justified) reputation for aloofness, showing that it is not out of touch with the values espoused by many city residents. The mayor’s participation in May 2017 street march organized in support of gay rights was seen as a step in the right direction (regrettably, the mayor was murdered in January 2019). The same can be said about the authorities’ decision to set up a council for equal treatment (e.g., homosexuals) and a council for immigrants.

## Conclusions

By placing the discussion within a theoretical framework that combines stakeholder and constraints theories, this paper provides important insights at the interface between urban scholarship, political science and management studies. Its major contributions include the use of constraints theory for analysis of participatory processes and the conceptualization of the relationship between a stakeholder and a municipality-owned company as nested and location-dependant. The findings add substance to the view that exploration of individual motivation to get engaged in urban management should occupy a prominent place in the study of participatory governance and (public-sector) managerial practices in cities. After all, even well-designed participatory arrangements and deliberative mechanisms might be of little use if city residents are reluctant to participate. Our study also demonstrates applicability of a specific quantitative methodology to analysis of urban-governance phenomena. Last but not least, by drawing on data collected in Poland, this research work responds to calls for studying this mode of governance in cultural contexts other than the Americas and Western Europe.

As in the vast majority of research projects, this study has a number of limitations that ought to be acknowledged, all of which provide opportunities for further research. First, it relies solely on self-reports, which suggests that caution is in order while interpreting and generalizing the findings. Second, although our sample represents, from a socio-economic perspective, a diverse population, it is not geographically diverse. Future researchers might wish to collect data from stakeholders living elsewhere in Poland or other parts of Eastern Europe. Third, the study is cross-sectional and correlational in nature. Therefore, our analysis identifies correlations rather than causation. It follows that, given that a longitudinal design is required to establish cause-and-effect associations, one might carry out the same questionnaire survey in, say, two years’ time. Fourth, it is essential to remember that motivation is a complex psychological phenomenon, which implies that other factors might come into play when exploring stakeholder willingness to participate. For example, self-determination theory [79] holds that individuals’ motivations can come from both intrinsic factors such as curiosity and values, and extrinsic factors such as pressure and rewards. Unfortunately, in our questionnaire, we did not ask respondents to declare their personal values or ideological convictions. Thus, future researchers, while preparing their questionnaires, could include a construct called “personal values” in an attempt to ascertain whether these are correlated with one’s intention to participate in urban managerial processes. Relatedly, it might also be interesting to explore the implications of deterrence theory, which “envisions people as rational maximisers of self-interest, responsive to the personal costs and benefits of their choices, yet indifferent to the moral legitimacy of those choices” [80], for the question of civic engagement. Fifth, we did not control for individual income (i.e., for legal and cultural reasons), which, to repeat, could have allowed us to find out whether low earners are less likely to get involved in civic life than higher-earning residents. Sixth, we drew on a simplified conceptualization of constraints theory. Thus, future researchers might consider using structural as well as interpersonal and intrapersonal constraints. Seventh, we did not manage to interview either MTG managers or representatives of the city government with a view to finding out how they conceptualize stakeholder participation and empowerment. It could, therefore, be informative to interview them on these fundamental issues in the future. Finally, one might argue that this study’s population represents an accidental collection of individuals, some of whom may not even be aware that they are stakeholders of MTG. Yet this criticism would miss the point since one of our principal goals was to ascertain whether ordinary people (i.e., residents of Gdansk in general and residents of Letnica in particular), among others, are aware that they have a stake in the undertakings that shape their local space, with all the resulting implications for their civic engagement (i.e., this is all the more so given that the construction of AmberExpo was well publicized by local media). These limitations notwithstanding, we believe that this study advances our understanding of the socio-psychological mechanism underlying participatory processes and managerial practices in cities, at the same time reigniting the debate over the focus and method of inquiry in social science.

## Supporting information

S1 Table Relative frequencies for controls of the groups of residents (%). (DOCX)
