# Supplementary material for: Participatory urban governance: Multilevel study
Source: PLoS One. 2020 Feb 21;15(2):e0229095. doi: 10.1371/journal.pone.0229095 (PMC7034860; doi:10.1371/journal.pone.0229095)
Supplement: S1 Table — (DOCX) [file pone.0229095.s001.docx]

**S1 Table. Relative frequencies for controls of the groups of residents (%).**

| Group | Sex | | Age | | | | Education | | |
| --- | --- | --- | --- | --- | --- | --- | --- | --- | --- |
|  | F | M | < 25 | 26 - 40 | 41 - 65 | > 65 | Primary | Secondary | Higher |
| G4 | 57 | 43 | 26 | 18 | 38 | 19 | 15 | 46 | 40 |
| G5 | 45 | 55 | 27 | 17 | 23 | 34 | 34 | 44 | 22 |
| G6 | 40 | 60 | 44 | 0 | 0 | 56 | 64 | 36 | 0 |
| G7 | 63 | 38 | 32 | 53 | 15 | 0 | 3 | 41 | 56 |
